# Dr. Taber and the Cyclopædia of Drug Pathogenesy

**Published:** 1887-11

**Authors:** Richard Hughes

**Affiliations:** Brighton, England


### DR. TABER AND THE CYCLOPÆDIA OF DRUG PATHOGENESY.

Richard Hughes, M. D., Brighton, England.

As I am personally responsible for the presentation of Dr. Taber's symptoms of Picric acid in the *Cyclopœdia*, I must ask space for a few words in reply to his criticisms thereon contained in the August number of The Homœopathic Physician.

The work was accomplished under considerable difficulty. Learning from Dr. Allen that he had returned to Dr. S. A. Jones the MS. of his provings, we wrote to the latter for the loan of them, but received no reply. It was necessary, therefore, for our purpose to reconstruct the pathogeneses of his experimenters from the symptoms dispersed through Dr. Allen's schema. I am not conscious of any want of care in so doing, and, while thankful for any correction, am rather relieved to find that so little is needed.

Dr. Taber made three provings of the acid, and finds fault with our transcription of the third only. Of the errors he notes, one is a misapprehension of his own; our "3 d." does not mean "third day," but "three days." Another—the "morning micturition" of the fifth day—is confessedly to be laid at Dr. Allen's door. A third—tenth for eighteenth day—is a printer's mistake, naturally passed over in correction. Of those that remain I can only take blame for the omission of the concomitant of the occipital headache on the fourth day and the error of half an hour on the third day. The account of the dosage is, for all practical purposes, correct. The symptom of the second day was omitted as too trivial for record. "Cardiac orifice" seemed the best equivalent for the very vague "cardiac region of the stomach," mentioned by Dr. Taber. The "all day" of the fifth day was a misunderstanding, which I am glad to have set right, but I submit that a pain which comes on

"while walking a short distance" may fairly be described as occurring "after a short walk."

If these corrections had simply been furnished as a friendly contribution to the perfecting of the *Cyclopædia*, I should have said nothing, save to express our thanks for them when incorporating them into the appendix to volume two. But when Dr. Taber puts them forward as grounds for impugning the faithfulness of our work, I am compelled, in its interests, to subject them to analysis, and I think it will be acknowledged that the result does not warrant his inference.

While we eagerly invite emendations from every source, we would suggest that authors of provings which have not yet been printed in detail should send us their MS. that we may have them at first hand, and so be spared such mistakes—however few—as arise during the difficult process of reconstructing them from a schema.*

* I observe that Dr. Kent speaks of being "unable to see any value" in the *Cyclopædia* or to "gather assistance" from it. While I regret this, I can hardly be surprised; as, judging from his published lectures, Dr. Kent seems to practice mainly upon empirical indications, which, useful as they are, play no part in the method of Hahnemann, and, of course, need no pathogeneses. It is for those who wish to carry out that method in its integrity, fitting drug-action to disease on the principle *similia similibus*, that the *Cyclopædia* is constructed, and I feel sure that they will appreciate its value.

